# Vestibular Deficits in Neurodegenerative Disorders: Balance, Dizziness, and Spatial Disorientation

**DOI:** 10.3389/fneur.2017.00538

**Published:** 2017-10-26

**Authors:** Thomas Cronin, Qadeer Arshad, Barry M. Seemungal

**Affiliations:** ^1^Division of Brain Sciences, Department of Medicine, Charing Cross Hospital, London, United Kingdom

**Keywords:** vestibular system, vestibular perception, neurodegeneration, spatial disorientation, balance

## Abstract

The vestibular system consists of the peripheral vestibular organs in the inner ear and the associated extensive central nervous system projections—from the cerebellum and brainstem to the thalamic relays to cortical projections. This system is important for spatial orientation and balance, both of critical ecological importance, particularly for successful navigation in our environment. Balance disorders and spatial disorientation are common presenting features of neurodegenerative diseases; however, little is known regarding central vestibular processing in these diseases. A ubiquitous aspect of central vestibular processing is its promiscuity given that vestibular signals are commonly found in combination with other sensory signals. This review discusses how impaired central processing of vestibular signals—typically in combination with other sensory and motor systems—may account for the impaired balance and spatial disorientation in common neurodegenerative conditions. Such an understanding may provide for new diagnostic tests, potentially useful in detecting early disease while a mechanistic understanding of imbalance and spatial disorientation in these patients may enable a vestibular-targeted therapy for such problems in neurodegenerative diseases. Studies with state of the art central vestibular testing are now much needed to tackle this important topic.

## Introduction

The peripheral vestibular apparatus transduces head acceleration, both angular and linear acceleration (including gravity being equivalent to a physical linear acceleration), and functionally speaking plays an important role in the control of eye movement, posture, gait, and egocentric perception. The vestibular end-organ consists of the otolith organs—which transduce linear acceleration; and the semi-circular canals which transduce angular acceleration. From there the signal pass to the vestibular nerve, the brainstem and cerebellar circuits, vestibular thalamic projections, vestibulospinal projections, and finally the vestibular cortical network. Functionally, the vestibular system senses self-motion (“am I moving”) and spatial orientation (“where am I now”), with the neuroanatomical correlates mediating these vestibular sensations being distinct.

Vestibular, visual, and proprioceptive sensory input is integrated in the brain and used to subsequently adjust the outgoing motor response to maintain balance, posture, and gaze stabilization. Vestibular dysfunction—arising from peripheral or central components of the vestibular system—may manifest as illusory self-motion (dizziness/vertigo) and spatial disorientation, which in turn can impair balance.

The overall prevalence of vestibular dysfunction in adults aged over 40 in the USA is 35%, representing 69 million individuals (1). Specifically, patients with vestibular dysfunction are at significantly greater risk of falls, involving both symptomatic and asymptomatic patient groups (1). Resultantly, vestibular dysfunction has a major impact on mortality, morbidity, health-care resources (1), and socioeconomic productivity (2).

The vestibular system is phylogenetically one of the oldest of all of the sensory systems (3) and the earliest to mature during development (4) and, thus, presents a potentially pertinent area of study in the context of neurodegeneration. For instance, work in Alzheimer’s disease (AD) demonstrates areas of neuronal degeneration in phylogenetically older neurones (5).

With an aging population, we are facing a rise of both vestibular disorders and neurodegenerative conditions. Dizziness and imbalance are important in neurodegenerative disease due to their association with falls. In addition, detection of dizziness and imbalance, and specific vestibular testing may have a potential role in the identification of neurodegeneration, especially in the initial stages of the disease process. For future treatments in neurodegenerative disorders to be most effective, earlier detection is likely to be vital to restrict neuronal loss.

In this review, we will focus on dizziness, imbalance, and spatial disorientation in relation to the neurodegenerative conditions of AD, progressive supranuclear palsy (PSP), frontotemporal dementia (FTD), motor neurone disease (MND), multiple system atrophy (MSA), and Parkinson’s disease (PD). In doing so, we demonstrate how detecting central and even peripheral vestibular pathology is important for the diagnosis and management of these conditions.

## Alzheimer’s Disease

Alzheimer’s disease is a from of dementia that typically presents with memory disturbance. It is characterized by beta-amyloid deposition in the brain, neurofibrillary tangles, and neuronal death. Imbalance is a little recognized feature of AD (6, 7), despite the condition carrying a high risk of falls (8) and gait abnormalities (9). Up to third of newly diagnosed AD patients complain of spatial disorientation (10), with wandering, for instance, a frequent symptom of AD (11).

Patients with AD are also three times more likely to experience a fracture compared to age-matched controls (12), and 47% have been observed to fall over the course of 1 year (13). Moreover, falls have been suggested to precede detectable cognitive changes in AD patients. A prospective study of presumptive preclinical AD patients found a higher rate of falls compared to aged-matched controls (14).

Nakamagoe et al. evaluated balance in individuals with AD and healthy aged-matched controls, using an eyes-closed stepping test. They reported that AD subjects were significantly more likely to move and turn than stay in the same position after 50 steps (7). Furthermore, stabilometry (objective study of body sway during quiet standing) testing has been demonstrated to be significantly altered in AD patients across measures of antero-posterior sway, latero-lateral sway, and area of confidence ellipsis, worsening in each parameter when eyes were closed. In particular, the authors identified a strong correlation between impairment in the anterior–posterior sway component for the AD subjects and reduced cognitive scores (6). This has led some researchers to suggest balance disturbance is the leading cause of falls in patients with AD (7).

Further insights into the cause of falls in AD patients have been identified by provoking compensatory postural adjustments through virtual reality (15). Patients with AD demonstrated slower response times to adjusting body position in response to changing visual stimuli, with this effect pronounced in the AD group with a history of falls. The AD faller group was also shown to have abnormal postural correction, reflecting worse inherent postural stability. Postural control was also related to higher cognitive processing, with the authors concluding that falls may result in AD patients from insufficient cognitive resources to control posture. Indeed, dual-task gait testing (assessing gait while performing a challenging cognitive task) in patients with mild cognitive impairment who went on to develop dementia showed a reduced gait performance relative to those who did not (16), with future study addressing this paradigm in other neurodegenerative disorders (17).

This follows from work performed by Barra and colleagues that used spatial and verbal tasks in conjunction with a balance task performed in young healthy adults which found an increase incidence of falls during spatial-task performance. The authors concluded that cognitive performance was maintained at the expense of balance, transgressing the “posture first” principle (18). Note, other studies have suggested a principle of “posture first” in older adults and “cognition first” in younger adults (19); however, exactly why, when, and what causes this change in strategy remains obscure.

A core brain area implicated in spatial orientation is the hippocampus (20), with this area being among the earliest regions to degenerate during the course of AD (21). Aside from a few studies within humans (22, 23), the evidence for a major role of the vestibular system in hippocampal function has come from animal studies involving vestibular stimulation or lesions (24–27). Further, in a study by Brandt et al., they demonstrated that in patients with bilateral vestibular loss, major atrophy of the hippocampus that correlated with impairments on spatial memory tasks (23). This association has led some to speculate on causal relationship between peripheral vestibular loss and AD (28, 29). Namely, anterograde degeneration, in which destruction of lower structures, i.e., peripheral vestibular apparatus, leads to degeneration of their higher projection zones, i.e., vestibular projections and the hippocampus (28). However, there is no empirical epidemiological evidence to support peripheral vestibular loss as a risk factor for AD.

A related but distinct question is the role of the hippocampus in vestibular cortical processing. Over the last four decades, animal experimentalists have demonstrated the remarkable properties of a group of cells in the hippocampus—place cells—that effectively indicate the position of the animal within its environment (30). These cells’ indication of spatial position are updated by vestibular input, especially in the dark, and indeed, vestibular ablation renders these cellular systems permanently dysfunctional (31), indicating that the integrity of the peripheral vestibular system is obligatory for these spatial guidance systems.

A key concept in place cell functions (and head direction cells that provide a compass like indication of head angular orientation) is the conversion of inertial signals of motion to position—a function called path integration (30). Two recent human lesion studies, however, found no effect of hippocampal lesions upon path integration function (32, 33). Instead, lesions, due to stroke, in the temporoparietal junction (TPJ) (33) have been shown to impair vestibular-guided spatial orientation. It follows that AD may affect spatial orientation by its effect on vestibular cortical regions such as the TPJ.

Perhaps more important is the impact upon cortical networks with multimodal imaging studies showing a consistent disruption in AD (34). Given the evidence of a widespread vestibular brain network involved in the vestibular perception of self-motion (35, 36), it can be expected that pathological changes associated with AD are likely to impact upon this neural system.

## Progressive Supranuclear Palsy

PSP is a pathologically defined disease underpinned by the accumulation of hyperphosphorylated tau throughout the brain, as well as in distinctive regions. Its clinical phenotype is however variable. PSP often presents with falls early in the course of the disease (37, 38). The midbrain is affected early on in the disease course (39). Although the vestibular nuclei (primarily in the pontomedullary junction) show loss of neurones at autopsy (40), the angular VOR (dependent on the semi-circular canal system and producing eye rotations to compensate for head angular rotation) is relatively maintained until later stages of the disease course (41), inferring preserved canalicular projections. In contrast, failure of saccular projections to the vestibular nuclei result (39) in markedly impaired linear (translational) VOR—a function reliant upon the otolithic sacculus and utriculus (42). This otolihic dysfunction corresponds clinically with the impaired ability of PSP patients’ convergence and near viewing of a target, and may also reflect damage to the interstitial nucleus of Cajal (43, 39).

The hypothesis of saccular projection impairment is further supported by vestibular-evoked myogenic potentials (VEMPs) testing in patients with PSP. cVEMPs consist of inhibitory potentials recorded from the sternocleidomastoid (“cervical” VEMP—cVEMP) in response to loud sounds, and are used in the testing of vestibulospinal reflexes. During movement, otolithic inputs are integral for producing the vestibulospinal reflexes that adjust muscle tone so that stable posture can be maintained. Depending on bone or air sound conduction, saccular afferents can be preferentially activated through cVEMPs, with this being the case in the latter conduction (44). In contrast, oVEMPs uses the inferior oblique muscle of the eye (“ocular” VEMP—oVEMP) to measure utricular function (45). Liao and colleagues found a significant reduction of cVEMP amplitude in PSP patients compared to age-matched healthy control group, with air sound conduction, inferring impaired function of the saccular pathways (42). They concluded that since the pathways mediating cVEMPs synapse in the lateral vestibular nuclei, this was not necessarily an inevitable feature of degenerative brainstem disease, but rather a specific sign in PSP.

Accordingly, the impaired ability to adjust vestibular reflexes for translational motion through the environment may be one component in the postural defect in PSP (42). However, how much this contributes to postural instability and falls in comparison to other factors is yet to be elucidated. Indeed, findings of impaired proprioceptive sensory inputs in PSP indicate it is likely to be an abnormality in central sensory integration, rather than a sole vestibular deficit (46). Dale et al. performed postural stability tasks on PSP patients versus healthy controls, finding patients with PSP had an inability to perceive backward tilt of the surface or body. Proposals for future study are focusing on the association between the VOR, postural deficits and falls in PSP (47).

Chen et al. have related this possible PSP pathogenesis ecologically to the bipedal upright locomotion (39). They proposed that PSP may owe its selective set of disturbances of eye movements and balance due to restricted involvement of a recently developed neural system that deals with erect permanent bipedal locomotion, the main components of which lie in the midbrain. Nevertheless, a distinct neural system for bipedalism is contentious, and furthermore, permanent bipedalism can be considered an adaptation of what is common—intermittent bipedalism, and whether a neural system between these states is distinct would be a further level of speculation. It is unclear whether PSP affects primates, although recent neuropathological analysis of cynomolgus monkeys found the cytopathology and distribution of tau deposits resemble those of PSP (48).

Additional vestibular mechanisms that may contribute to postural instability in PSP may include the vestibulo-collic reflex, which stabilizes the head on the body. PSP patients often show head turns opposite to the direction of intended gait due to over activity of the vestibulocollic reflex (49), which has been notionally attributed to the involvement of the brainstem reticular nuclei (50).

Computerized posturography testing can differentiate early PSP from early PD (51) and age-matched controls (52). Ondo and colleagues utilized the sensory organization test (SOT), where subjects are asked to stand still under a variety of altered sensory conditions (51). The SOT parameter that best differentiated PSP and PD was when both visual and proprioceptive inputs were deprived, leading the authors to conclude there was a vestibular pattern of dysfunction.

The limit of stability test (LOS) was also found to be abnormal in PSP (51, 52). LOS measures path sway, time, and distance traveled by the patient’s center of gravity from an initial starting point to eight different points (51). The backward direction score was identified as being most severely affected, which is consistent with the higher frequency of falls in the backward direction in PSP patients (52). Of note, preservation in scores for the left (non-dominant side—with testing being performed on right sided dominant individuals) and forward-left (non-dominant forward diagonal) directions were reported (52) and may reflect the distribution of central PSP pathology.

This backward fall phenomenon may draw comparison with “Tumarkin” drop attacks (“otolithic crises”) found in a subset of Meniere’s disease patients. Tumarkin falls occur without warning and without loss of consciousness, with a stereotyped direction, bearing similarities to falls in PSP. The pathophysiology of Tumarkin drop attacks is felt to be caused by a burst of neural impulses from the otolithic organs to the vestibulospinal pathways, triggering the fall (53). Indeed, cVEMP testing in Tumarkin patients has demonstrated which were more likely to be elevated or absent thresholds compared to the patient’s unaffected ear, implicating the involvement of the saccule in these patients (54). Similarly, as mentioned earlier, cVEMP measurements are also found to be abnormal in PSP (42), although this is likely to implicate saccular projections, rather than peripheral dysfunction as in Tumarkin attacks. Furthermore, falls in PSP are likely to be multifactorial, with axial rigidity also likely contributing to the nature of PSP falls (55).

Studied techniques to improve balance in PSP have involved audio-biofeedback (56). This consists of adding artificial sensory information that informs the brain about actual body posture and movements. In a study of eight patients with PSP, significant improvements in the Berg Balance Scale (which involves 14 different balance tasks) were observed after 6 weeks.

## Frontotemporal Dementia

Frontotemporal dementia is characteristically a pre-senile dementia that presents with a progression deterioration of personality, social interaction, and cognition. Studied measures of gait and balance have been found to be abnormal in FTD when compared with controls (57). The limit of stability and dynamic balance testing were impaired in patients with FTD. In comparison, spatial orientation has been found to be relatively intact in FTD individuals (58). Tu and colleagues investigated spatial orientation using a novel virtual supermarket task to compare patients with AD and FTD. Subjects watched a sequence of videos from a first-person perspective moving through a virtual supermarket and were commanded to preserve orientation to an initial starting point. Analyses revealed significantly impaired spatial orientation in AD, compared to FTD patient groups, and was able to discriminate the two groups to a high degree at presentation.

Voxel-based morphometry, a neuroimaging analysis technique to investigate focal differences in brain anatomy, was also performed on the subjects, identifying significantly greater atrophy in medial parietal and retrosplenial regions for AD patients compared to FTD patients. The authors went on to speculate that the retrosplenial region plays a crucial role in spatial orientation (58).

Nakamagoe et al. performed caloric and visual suppression testing on 14 patients with FTD (59). In healthy subjects, vestibular-nystagmus induced by the caloric test can be suppressed by visual fixation (i.e., visual suppression) and impaired visual fixation is typically indicative of a central pathology (i.e., cerebellum, brainstem and cerebral cortex). It was found that FTD participants typically had an impaired visual suppression compared to controls. Further analysis was performed according to clinical features of the FTD patients, indicating that visual suppression of the VOR was significantly more altered in FTD patients with gait disturbance. The authors concluded that damage to the vestibular cortex, which they related to the inferior parietal lobule, might be responsible for the impairment of visual suppression in FTD patients. However, one caveat to this interpretation would be the identification of a discrete vestibular cortex, rather than the notion of distributed central projections of vestibular information in various cortical networks (60, 61).

## Motor Neurone Disease

Motor neurone disease is a progressive disorder in which degeneration of the upper and lower motor neurons leads to progressive weakness of bulbar, limb, and trunk muscles. As a result, falls are common in patients with MND, with a prospective longitudinal cohort study of MND patients showing an annual incidence of 64% (62). Interestingly, a study of head and other physical trauma injury in patients with MND, demonstrated a higher risk of injury compared to controls in the first year after diagnosis that subsequently reverted back to the level in the control group 1 year after diagnosis (63). In addition, with many MND patients reporting unsteadiness and fear of falling early in the course of the disease (64), this may suggest other factors as well muscle weakness may be contributing to falls in MND.

Sanjak et al. used SOT in computerized posturgraphy to assess vestibular deficits in patients who were ambulatory with MND compared to healthy controls (64). They found that MND subjects in the normative range in clinical mobility displayed distinct impairment in equilibrium testing and an increased number of falls during conditions of altered support surface, when vision was absent or sway-referenced, in comparison with healthy controls, suggesting a vestibular pattern of impairment. The authors hypothesized that cerebellar involvement in MND may result in this particular pattern of vestibular deficit since the peripheral function was preserved in these patients. Nonetheless, caution is required on the interpretation of SOT in such patients, when factors such as inherent muscle weakness and spasticity may also lead to postural instability and increased body sway, independent of vestibular dysfunction.

Vestibular-evoked myogenic potential measurement has also been performed on patients with MND, showing no abnormalities in patients in the early stages of the disease (65). Additional assessment of the vestibular system in MND has found abnormalities in visual suppression (66) and caloric testing (67). Ohki et al. found abnormalities of visual suppression in two out of nine patients with early stages of MND (66), which is indicative of cerebellum pathology.

## Multiple System Atrophy

Multiple system atrophy is an a oligodendrogliopathy characterized by prominent alpha-synuclein inclusions, resulting in neuronal death, which manifests clinically with autonomic failure, ataxia, and parkinsonism. Balance and gait are also frequently found to be disturbed in MSA (68), and symptom assessment scales focusing on these parameters are important for the evaluation of patients in early stages of MSA (69).

It is typically classified into a cerebellar predominant (MSA-C) and parkinsonism predominant (MSA-P) subtypes. Lee and Koh retrospectively identified the clinical features of 20 MSA-C patients, with disequilibrium (50%) and dizziness (15%) the most common initial presentation (38). For 21 MSA-P patients, tremor was the most frequent symptom (19%), while dizziness was found in 10%. Similarly, Sakakibara and Hirumab found 60% (9/15) of patients with MSA-C reported dizziness on head-turning (70).

Falls are frequent in MSA (71), and abnormalities in VEMPs for MSA patients have been associated with an increased risk of falling (72).

It is important to note, however, due to the frequent finding of orthostatic hypotension, identifying vestibular-related dizziness and balance impairment can be a challenge (73, 74). Nevertheless, vestibular function testing in MSA is abnormal (72, 75), pathological studies at autopsy show neuronal loss in the vestibular nuclei (76) and neuroimaging demonstrate degeneration in flocculus and nodulus in the cerebellum of MSA patients (70).

Impaired VOR suppression on visual fixation has also been identified in MSA (70, 77, 78). Indeed, its use as has been put forward as a method of distinguishing PD from MSA (78). Despite this assertion, abnormalities have been found in the cerebellum of PD patients (79), and impaired VOR suppression has been documented in such patients (80).

## Parkinson’s Disease

Parkinson’s disease is broadly classified as a “movement disorder” but encompasses a wide variety of motor and non-motor symptoms, which results from the irreversible loss of dopaminergic neurones. Postural instability is one of the most disabling features in PD. Using computerized posturography integrated with a virtual reality system to analyze LOS, patients with PD were found to have a reduced LOS area and greater postural sway compared with healthy subjects (81). The deterioration in postural control was significantly associated with major risk of falls. Additionally, the manipulation of sensory input on the subjects was suggestive of reduced use of vestibular information to maintain postural control. Moreover, computerized posturography using SOT in patients with PD also demonstrated impaired processing of vestibular information (82, 83), with additional study indicating this was independent from the stage of the disease (84).

Perturbation of proprioceptive information in PD patients found no reweighting of vestibular inputs (85), which contrasts when performed in healthy subjects (86), with authors concluding that the issue of postural control in PD lay not in the ability to generate movement but the inability to perceive movement. However, this conclusion, neglects the issue of impaired anticipatory postural adjustments found in PD, while the sensory evaluation performed was limited to visual, vestibular, and proprioceptive stimulation.

Functional neuroimaging of PD patients has demonstrated reduced neuronal activity in the cingulate sulcus visual area (87), where vestibular and optic inputs are integrated (88), as well as showing reduced activation of this area is associated with increased disease severity (87). Therefore, a deficit of central sensory processing in PD is implied.

Vestibular-evoked myogenic potential responses in PD patients have been linked to the motor and non-motor symptoms (89). Specifically, impaired cVEMP testing in PD patients has been shown to be correlated significantly to contralateral rigidity, bradykinesia severity, ipsilateral dyskinesia scores, as well as sleep, mood, and memory impairment. Indeed, cVEMP testing in PD patients compared to aged-matched controls has been frequently found to be abnormal (72, 90, 91). This reflects potential brainstem pathology among PD patients, which links previous study of pathological changes in the vestibular nuclei of PD patients (92), and disrupted connections between vestibular nuclei and the dorsal raphe nuclei (93). Additional mechanisms for this may include the reduced effect of dopamine on the excitability of vestibular nuclei found in PD patients (94).

Peripheral ipsilateral vestibular paresis has been associated with lateral trunk deviation (Pisa syndrome) in patients with PD (95). In addition, the perception of the subjective visual vertical (the ability of a person to perceive earth-vertical with respect to gravity) has been demonstrated to be deviated in PD patients with lateral trunk flexion (96). Gandor and colleagues produced a similar finding in PD patients, and discussed that altered verticality perception in PD may reflect a central vestibular processing deficit (97).

The symptom of freezing of gait (FOG) in PD has also been related to the vestibular system. Huh et al. evaluated PD patients with FOG, PD patients without FOG, and aged-matched healthy controls using the SOT (98). PD patients with FOG showed worse postural sensory processing compared to those without FOG and a particular inability to use vestibular information. The authors attributed this with abnormal central processing of vestibular signals in PD. However, a causal relationship between FOG and impaired vestibular processing based on these results cannot yet be established until future research analyzing the imaging correlates of postural sensory deficits in PD patients with FOG is undertaken.

The brain area implicated in FOG is the pedunculopontine nucleus (PPN) (99). Direct projections to the PPN from vestibular nuclei have been confirmed in primates (100), and vestibular stimuli in macaque monkeys enhance the activity of the PPN neurones (101). PPN deep brain stimulation (DBS) in PD reduces falls (102). We showed that PPN DBS in PD patients showed improved vestibular perceptual thresholds (103). Paradoxically, PPN stimulation worsened sway in these patient in the dark. Although this could imply worse postural control, a strategy of increased postural movement to improve sensory feedback could provide additional information to the vestibular system to help control balance. The recent developments in new DBS targets in improving balance control in PD provide a fertile ground for future study and therapeutic approaches, e.g., recently studies of PPN stimulation in PSP patients has showed promising results (104).

Similarly, targeted vestibular rehabilitation and therapy in PD has received attention, demonstrating improved postural control and balance performance (105–108). Stimulation of the vestibulospinal tract through proprioceptive disturbance and visual suppression improved double stance gait performance in patients with PD compared to those receiving standard physiotherapy (109). Moreover, in a single patient study, repeated caloric stimulation produced improvement in assessment scores for motor and non-motor symptoms of PD, which was sustained at 5-month follow-up (110). Yet, vestibular rehabilitation tended represents different techniques in different studies illustrating it as a potentially disparate practice. Furthermore, small number studies and frequent lack of randomization and comparator impair meaningful results.

Galvanic vestibular stimulation, involving transcranial direct current stimulation, can stimulate and inhibit vestibular afferents. Its use in PD patients has demonstrated improvement of postural instability (111, 112) and motor performance (113, 114). Similarly, stochastic vestibular stimulation, which uses subthreshold electrical noise has demonstrated improvements in postural control for PD patients (115, 116). These are, however, small number studies with limited follow-up of patients.

## Conclusion

This review highlights the role of vestibular function and dysfunction, in a number of neurodegenerative diseases, with a particular focus on the central vestibular system. Permanent bipedal locomotion is a hallmark of the human species, and is critically dependent upon the integration and processing of multiple sensory information (i.e., visual proprioceptive and vestibular sensory inputs), notwithstanding the requisite peripheral function. As a result, only limited neurodegeneration in central vestibular areas may result in significant clinical manifestations, especially imbalance and falls. Some of the disease areas discussed illustrate genuine advances in our understanding of neurodegenerative conditions, which can aid diagnostic and treatment strategies. A deeper mechanistic understanding of the role of the dysfunction of central vestibular systems in neurodegenerative disease is, therefore, much warranted.

Presently vestibular testing in neurodegenerative disease all too often focuses on peripheral (i.e., canal and otolith) function. Rather, testing should explore additional deficits in the central vestibular circuits. Indeed, state of the art exploration of central vestibular deficits is much warranted to provide a deeper mechanistic understanding of how balance and spatial disorientation so frequently arises in neurodegenerative disease.

## Author Contributions

All authors listed have made substantial, direct, and intellectual contribution to the work and approved it for publication.

## Conflict of Interest Statement

The authors declare that the research was conducted in the absence of any commercial or financial relationships that could be construed as a potential conflict of interest.
